# An actin cytoskeletal barrier inhibits lytic granule release from natural killer cells in patients with Chediak-Higashi syndrome

**DOI:** 10.1016/j.jaci.2017.10.040

**Published:** 2018-09

**Authors:** Aleksandra Gil-Krzewska, Mezida B. Saeed, Anna Oszmiana, Elizabeth R. Fischer, Kathryn Lagrue, William A. Gahl, Wendy J. Introne, John E. Coligan, Daniel M. Davis, Konrad Krzewski

**Affiliations:** a Receptor Cell Biology Section, Laboratory of Immunogenetics, National Institute of Allergy and Infectious Diseases, National Institutes of Health, Rockville, MD; b Manchester Collaborative Centre for Inflammation Research, University of Manchester, Manchester, United Kingdom; c Electron Microscopy Unit, Research Technologies Branch, Rocky Mountain Laboratories, National Institute of Allergy and Infectious Diseases, National Institutes of Health, Hamilton, Mont; d Office of the Clinical Director, National Human Genome Research Institute, National Institutes of Health, Bethesda, MD

**Keywords:** Chediak-Higashi syndrome, lysosomal trafficking regulator, natural killer cell, cytotoxicity, cytotoxic lymphocyte, lysosomes, lytic granules, exocytosis, immune deficiency, actin cytoskeleton, ARM, Armadillo repeats, CHS, Chediak-Higashi syndrome, CI-MPR, Cation-independent mannose 6-phosphate receptor, CMFDA, 5-chloromethylfluorescein diacetate, CRISPR, Clustered Regularly Interspaced Short Palindromic Repeats, CTL, Cytotoxic T cell, EEA1, Early Endosome Antigen 1, EV, Empty vector, HEAT, Huntingtin, elongation factor 3 (EF3), protein phosphatase 2A (PP2A), and the lipid kinase TOR repeats, HLH, Hemophagocytic lymphohistiocytosis, ICAM-1, Intercellular adhesion molecule 1, LAMP, Lysosome-associated membrane protein, LYST, Lysosomal trafficking regulator, MTOC, Microtubule organizing center, NK, Natural killer, PSF, Point spread function, Rab, Ras-associated binding protein

## Abstract

**Background:**

Chediak-Higashi syndrome (CHS) is a rare disorder caused by biallelic mutations in the lysosomal trafficking regulator gene *(LYST)*, resulting in formation of giant lysosomes or lysosome-related organelles in several cell types. The disease is characterized by immunodeficiency and a fatal hemophagocytic lymphohistiocytosis caused by impaired function of cytotoxic lymphocytes, including natural killer (NK) cells.

**Objective:**

We sought to determine the underlying biochemical cause of the impaired cytotoxicity of NK cells in patients with CHS.

**Methods:**

We generated a human cell model of CHS using Clustered Regularly Interspaced Short Palindromic Repeats (CRISPR) technology. We used a combination of classical techniques to evaluate lysosomal function and cell activity in the model system and super-resolution microscopy to visualize F-actin and lytic granules in normal and *LYST*-deficient NK cells.

**Results:**

Loss of LYST function in a human NK cell line, NK92mi, resulted in inhibition of NK cell cytotoxicity and reproduced other aspects of the CHS cellular phenotype, including the presence of significantly enlarged lytic granules with defective exocytosis and impaired integrity of endolysosomal compartments. The large granules had an acidic pH and normal activity of lysosomal enzymes and were positive for the proteins essential for lytic granule exocytosis. Visualization of the actin meshwork openings at the immunologic synapse revealed that the cortical actin acts as a barrier for secretion of such large granules at the cell-cell contact site. Decreasing the cortical actin density at the immunologic synapse or decreasing the lytic granule size restored the ability of *LYST*-deficient NK cells to degranulate and kill target cells.

**Conclusion:**

The cortical actin and granule size play significant roles in NK cell cytotoxic function. We present evidence that the periodicity of subsynaptic actin is an important factor limiting the release of large lytic granules from NK cells from patients with CHS and could be a novel target for pharmaceutical intervention.

Natural killer (NK) cells form a subset of lymphocytes involved in innate protection against tumors and microbial pathogens.^1^ They also modulate the adaptive immune response through production of chemokines and cytokines and regulation of activity and survival of other immune cells, such as dendritic cells, macrophages, or T cells.^2^, ^3^, ^4^ NK cells are best known for their ability to mediate cytotoxic elimination of abnormal cells. The killing of target cells is a complex process, culminating in localized delivery of lytic granules to the cell-cell contact site (ie, immunologic synapse),^5^, ^6^, ^7^, ^8^, ^9^ where the granules must navigate through the cortical actin meshwork before they can fuse with the plasma membrane and release their content.^10^, ^11^

Lytic granules are lysosome-related organelles that contain, in addition to typical lysosomal proteins, several unique molecules, such as perforin, granzymes, Fas ligand, or TNF-related apoptosis-inducing ligand.^6^ These proteins are indispensable for NK cell cytotoxicity because their release results in induction of target cell apoptosis. Defects in lytic granule exocytosis by NK cells, resulting in failure to control the abnormal growth of overactivated lymphocytes or macrophages, are associated with serious and life-threatening histiocytic disorders and lysosomal storage diseases, including familial hemophagocytic lymphohistiocytosis (HLH; types 2-5), Hermansky-Pudlak syndrome (type 2), Griscelli syndrome (type 2), and Chediak-Higashi syndrome (CHS).^6^, ^12^, ^13^, ^14^, ^15^, ^16^

CHS (OMIM 214500) is a rare disorder characterized by oculocutaneous albinism, predisposition to bleeding, and dysregulation of the immune system.^17^, ^18^, ^19^ The majority of patients have HLH, a fatal hyperinflammatory syndrome with loss of leukocyte homeostasis caused by impaired cytotoxic activity of T and NK cells.^17^, ^19^, ^20^, ^21^, ^22^ The disease is caused by biallelic mutations in the lysosomal trafficking regulator gene *(LYST)*,^23^ resulting in the distinctive feature of the disease: the presence of giant lysosomes or lysosome-related organelles (eg, lytic granules, melanosomes, and platelet-dense granules) in numerous cell types.^12^, ^24^, ^25^, ^26^, ^27^, ^28^, ^29^, ^30^ *LYST* encodes a protein with several domains implicated in various aspects of vesicular trafficking, such as Armadillo/Huntingtin, elongation factor 3 (EF3), protein phosphatase 2A (PP2A), TOR kinase (ARM/HEAT); pleckstrin homology; Beige and Chediak-Higashi; and WD-40,^18^, ^23^, ^31^ but its exact function remains to be elucidated.

The lack of a thorough understanding of the molecular and cellular mechanisms underlying several histiocytic disorders greatly restricts treatment options. Furthermore, the limited availability of patient samples hinders progress in understanding those rare disorders, especially CHS. Because NK cells require functional lysosomes to mediate the killing of target cells and defective NK cell cytotoxicity appears to control the progression of hemophagocytic syndromes,^6^, ^21^, ^32^ NK cells are an important model for investigating basic mechanisms of disease in patients with CHS. Our recent study revealed that *LYST* mutations lead to a heterogeneous range of defects in NK cells related to lytic granule size or polarization and acquisition of endolysosomal markers, resulting in severely impaired cytotoxicity without affecting cytokine secretion.^33^ Understanding the mechanism or mechanisms responsible for defective exocytosis and, consequently, cytotoxicity of NK cells could provide a key factor to therapy of CHS and the syndrome-associated HLH.

Although a few animal models of CHS exist, none of them fully reproduces the human disease.^34^ Furthermore, although many of the fundamental immunologic principles can be applied from mouse models to human subjects, several significant differences exist between human and mouse NK cells, such as initial functionality and cytotoxicity, differences in translation and expression of lytic proteins (perforin and granzymes) or cell-surface receptors, and pathways regulating NK cell activation.^35^, ^36^, ^37^ Therefore we sought to create a human CHS model to determine the underlying biochemical cause of the impaired cytotoxicity in CHS cytotoxic lymphocytes.

Here we report generation of an NK cell model of CHS that mimics the cellular phenotype observed in patients with CHS with *LYST* mutations in the ARM/HEAT domain, along with characteristic large granules. We demonstrate that lytic granules in NK cells from patients with CHS are functional and that the defect in NK cell degranulation is caused by hindrance from the actin cytoskeleton at the immunologic synapse. Importantly, we show that the degranulation and cytotoxicity of NK cells from patients with CHS could be restored by modulating the cortical actin meshwork density at the immunologic synapse or by decreasing the size of enlarged granules in *LYST*-deficient NK cells, suggesting new possibilities for CHS therapy.

## Methods

### Antibodies

The following antibodies were used against perforin (dG9), lysosome-associated membrane protein (LAMP) 1–Alexa Fluor 488 (H4A3), CD3 (UCHT1), CD56 (HCD56), and NKG2D (1D11; BioLegend, San Diego, Calif); cation-independent mannose 6-phosphate receptor (CI-MPR; 2G11), dynein heavy chain, and Ras-associated binding protein (Rab) 27a (Abcam, Cambridge, United Kingdom); early endosome antigen 1 ([EEA-1] 14/EEA1; BD Biosciences, San Jose, Calif); cathepsin D, Rab7, and Rab9 (Cell Signaling, Danvers, Mass); LAMP1 (H4A3), LAMP2 (H4B4), granzyme B (GB7), Munc13-4, and Rab14 (D-5; Santa Cruz Biotechnology, Dallas, Tex); myosin IIA and actin (AC-15; Sigma, St Louis, Mo); vesicle associated membrane protein 7 (Synaptic Systems, Goettingen, Germany); LAMP2–Alexa Fluor 488 (H4B4) (eBioscience, San Diego, Calif); and perforin (Pf-344, for immunoblotting; Mabtech, Stockholm, Sweden).

### Patients and healthy donors

Patients with CHS enrolled in protocol 00-HG-0153 (NCT00005917) and provided written informed consent. The protocol was approved by the Institutional Review Board of the National Human Genome Research Institute. The diagnosis of CHS was based on clinical findings and confirmed by identification of giant inclusions within leukocytes on peripheral blood smears. Mutations in *LYST* were identified in all subjects (patients A:1 and A:2, c.4361C>A and c.5061T>A; patient B, c.7951G>T; and patient C, c.4862+1G>A and c.9706C>T).^22^, ^33^, ^38^, ^39^ Voluntary healthy donors were recruited at the Department of Transfusion Medicine, National Institutes of Health, with the donor's informed consent in accordance with the Declaration of Helsinki.

PBMCs were isolated from whole blood samples by using the standard Ficoll-Paque method. NK cells were isolated from PBMCs by using EasySep Human NK cell enrichment kits (STEMCELL Technologies, Vancouver, British Columbia, Canada), according to the manufacturer's protocol.

### Cells

NK cells isolated from healthy donors or patients with CHS were cultured in X-Vivo medium (Invitrogen, Carlsbad, Calif) with 10% human serum and 100 U/mL IL-2. NK92mi cells from an IL-2–independent NK cell line derived from the NK-92 cells by means of transfection with human IL-2 cDNA^40^ were grown in X-Vivo medium with 10% human serum. Human B lymphoblastoid 721.221 cells were grown in complete RPMI 1640 medium.

### Clustered Regularly Interspaced Short Palindromic Repeats constructs

The Clustered Regularly Interspaced Short Palindromic Repeats (CRISPR) type II system was used to facilitate *LYST* editing. The sequences targeting the region encoding the *LYST* ARM/HEAT domain in genomic DNA were designed by using E-CRISP Designer (version 4.2) and aligned against those present in the human genomic and transcript database to verify the specificity of *LYST* targeting. The oligomers were synthesized, annealed, and cloned into lentiCRISPRv2 (Addgene, Cambridge, Mass).^41^, ^42^ The lentiviral expression constructs were used to create lentiviral particles and infect NK92mi cells.^43^ All CRISPR constructs were evaluated for their ability to disrupt *LYST* and generate a CHS-like cellular phenotype. The construct targeting the 5′-GAAGACCTTATTGTAATGCTTGG-3′ sequence of *LYST* (exon 28; c.7567-7589) was considered optimal for gene disruption and chosen to generate the *LYST*-deficient cell line. Small interfering RNA targeting the 5′-GGTTGACAGATGCAAGGAATC-3′ (R1) or 5′-TGTCTCACTGTACGGTTTAAT-3′ (R2) sequence of Rab14 (Sigma) were used for Rab14 silencing.

### Cytotoxicity assay

NK cell cytotoxicity was evaluated by using the DELFIA assay (PerkinElmer, Waltham, Mass).^33^, ^43^

### Flow cytometry

For analysis of total protein levels of LAMP1, LAMP2, granzyme B, or perforin, NK92mi cells were fixed, permeabilized with Cytofix/Cytoperm buffer (BD Biosciences), and stained with either anti–LAMP1–Alexa Fluor 647, anti–LAMP2–fluorescein isothiocyanate, anti-perforin–fluorescein isothiocyanate, or anti–granzyme B–Alexa Fluor 647 mAb.

Delivery of granzyme B to target cells was assessed with the GranToxiLux Assay Kit (OncoImmunin, Gaithersburg, Md).^33^, ^43^

Cathepsin B activity was determined by using the Magic Red Cathepsin B Assay Kit (ImmunoChemistry Technologies, Bloomington, Minn), according to the manufacturer's instructions. Cells were pretreated with 200 nmol/L concanamycin A for 60 minutes at 37°C before commencing the assay to disrupt cathepsin activity.

For lysosomal pH determination, NK92mi cells were labeled with 1 μmol/L LysoSensor DND-189 for 30 minutes at 37°C. The cells were then washed, LysoSensor fluorescence was measured, pH values were determined from the standard curve. To construct the standard curve, cells were preincubated in phosphate-citrate buffer with specific pH values (4.0-7.0; 0.5 pH increment between samples) for 30 minutes at 37°C, followed by labeling with LysoSensor and plotting LysoSensor median fluorescence intensity values as a function of pH. Cells were pretreated with 200 nmol/L concanamycin A for 60 minutes at 37°C to neutralize lysosomal pH.

Data acquisition and analysis were done with FACSCalibur or the LSRII cytometer (BD Biosciences) and FlowJo software (version 10; TreeStar, Ashland, Ore).

### Granzyme B activity

Granzyme B activity in cell lysates was assessed by measuring the hydrolysis of the Boc-Ala-Ala-Asp-thiobenzyl ester substrate.^43^

### Granule isolation

NK92mi cell homogenization and lytic granule fractionation on a 10% to 40% discontinuous gradient of iodixanol were performed, as described previously.^43^

### Drug treatment

NK92mi cells were pretreated with 0.5 μmol/L latrunculin B or 1 μmol/L swinholide A for 30 minutes at 37°C. The cells were then washed in PBS and used in the assays either alone or mixed with target cells for 45 minutes at 37°C; target cells were not treated with the drugs, and the drugs were not present in the samples while performing the assays. Lenalidomide (1 μmol/L final concentration; Celgene, Summit, NJ) treatment was performed, as described previously.^44^

### Microscopy and image analysis

NK cells were left alone or mixed with 721.221 target cells at a 1:1 ratio for 20 minutes at 37°C in X-Vivo medium, followed by adherence to Excell adhesion slides for 10 minutes at 37°C. Cells were fixed and permeabilized by using Cytofix/Cytoperm buffer with 0.1% Triton X-100 and 1% BSA. The cells were stained with anti-LAMP1 or anti-LAMP2 antibody, followed by Alexa Fluor 647–conjugated IgG_1_-specific anti-mouse antibody, or with anti–CI-MPR, anti–EEA-1, or anti-VAMP7 antibody, followed by Alexa Fluor 647–conjugated anti-mouse antibody or with anti-Rab27a, followed by Alexa Fluor 568–conjugated anti-goat antibody; blocked with 5% normal mouse serum; and then stained with Alexa Fluor 488–conjugated anti-perforin antibody. Alternatively, the cells were stained with anti-pericentrin, followed by Alexa Fluor 488–conjugated anti-rabbit antibody or Alexa Fluor 568–conjugated phalloidin; blocked with 5% normal mouse serum; and then stained with Brilliant Violet 421–conjugated anti-perforin antibody. Cells mounted in ProLong Gold medium were visualized with a Zeiss LSM710 Axiovert 200M laser-scanning confocal microscope (Zeiss, Oberkochen, Germany) at room temperature. The images were obtained with a ×63 Zeiss Plan-Apochromat objective and Zeiss Zen 2012 software. Colocalization and granule distances to the immunologic synapse and microtubule organizing center (MTOC) were assessed by using Imaris software (version 8.0.1; Bitplane, Zurich, Switzerland), as described previously.^33^

NK cells were stained with anti-perforin, anti–CI-MPR, or anti-LAMP2 antibodies, as described above, and imaged in all 3 planes (x, y, and z; optical slices were collected at 0.3-μm intervals) to evaluate vesicle size and number. The acquired images were deconvolved with Huygens software (version 17.04; Scientific Volume Imaging B.V., Hilversum, The Netherlands) to correct image degradation caused by point spread function (PSF). PSF was obtained through distilling an experimental PSF from spherical bead images. Subresolution beads from the PS-Speck Microscope Point Source Kit (Invitrogen) were placed on Excell Adhesion slides, mounted in ProLong Gold medium, and imaged as described above, with the z-axial dimension of 0.1 μm to obtain calibration images of beads; PSF was reconstructed from the averaged bead images by using Huygens software, according to the Scientific Volume Imaging protocol. After obtaining the PSF, the iterative maximum likelihood estimation CMLE algorithm was used to effectively solve image convolution and perform deconvolution. Deconvolved images were analyzed with Imaris software by using the Spots function, with the following parameters: estimated diameter for the vesicle identification was set to 0.3 μm, spots were classified based on the quality criterion algorithm, region growing function was based on the local contrast, and spot diameter was determined from region border.^33^

For STED imaging, NK cells were added to the wells of chambered glass coverslips (no. 1.5 Lab-Tek; Thermo Scientific Nunc, Paisley, UK) coated with 0.01% poly-l-lysine and either recombinant intercellular adhesion molecule 1 (ICAM-1; 2.5 μg/mL; R&D Systems, Minneapolis, Minn) or recombinant ICAM-1 and MICA-Fc (2.5 μg/mL; R&D Systems).^10^ Cells were incubated at 37°C for 8 minutes, followed by fixation with 4% paraformaldehyde/PBS solution, and stained with Alexa Fluor 568–conjugated phalloidin and Alexa Fluor 488–conjugated anti-perforin antibody. STED images were obtained with a Leica TCS SP8 STED CW microscope equipped with a ×100 oil immersion lens (NA 1.4) at room temperature. Sequential scanning was applied during acquisition, and optical slices were collected at 0.22-μm intervals. STED images were deconvolved in Huygens Professional 17.04 software. Actin mesh periodicity was analyzed, as previously described.^44^, ^45^

### Cell conjugation

NK cells and 721.221 target cells were labeled with 2 μmol/L CMFDA (5-chloromethylfluorescein diacetate, Invitrogen) and TFL-4 (1:3000), respectively. Cells were washed 3 times in PBS with 2% FCS and mixed together at an effector/target ratio of 1:2. Cells were immediately transferred to 37°C and incubated for 30 minutes, followed by fixation with 1% paraformaldehyde and analysis with flow cytometry. For time 0, cells were mixed and immediately fixed. Conjugate percentages were established by determination of CMFDA^+^TFL-4^+^ double-positive cells, representing NK cells conjugated with target cells, from the total pool of live CMFDA^+^ NK cells.

### Transmission electron microscopy and immunotransmission electron microscopy

Cells were fixed with 2.5% glutaraldehyde in 0.1 mol/L Sorensen phosphate buffer (pH 7.4). After washing 3 times with 0.1 mol/L sodium cacodylate buffer (pH 7.4), cells were postfixed with 0.5% osmium tetroxide and 0.8% potassium ferricyanide in 0.1 mol/L sodium cacodylate buffer for 1 hour at room temperature. After 3 more buffer washes, cells were stained with 1% tannic acid in dH2O for 1 hour, rinsed 3 times with dH2O, and then further stained with 1% uranyl acetate overnight at 4°C. After 3 additional dH2O washes, cells were dehydrated with a graded ethanol series through 3 washes with 100% ethanol. The cells were then infiltrated and embedded with Spurr resin and cured overnight at 68°C.

For immunotransmission electron microscopy, cells were fixed in periodate-lysine-paraformaldehyde fixative with 0.25% glutaraldehyde. After washing with PBS, they were labeled for an hour with primary (anti-LAMP1 or anti-perforin) and secondary (anti-mouse IgG–horseradish peroxidase) antibodies in 0.05% saponin in PBS. Cells were then fixed in 1.5% glutaraldehyde in 0.1 mol/L sodium cacodylate with 5% sucrose, washed in 50 mmol/L Tris-HCl with 7.5% sucrose (pH 7.4), and labeled with metal enhanced DAB substrate (Thermo Fisher Scientific, Waltham, Mass). After immune labeling, cells were fixed with 2.5% glutaraldehyde in 0.1 mol/L sodium cacodylate (pH 7.4) overnight at 4°C and subsequently postfixed for 1 hour in 1% osmium tetroxide and 0.8% potassium ferricyanide in 0.1 mol/L sodium cacodylate buffer at room temperature. After 3 washes with dH2O, cells were dehydrated with a graded ethanol series and then infiltrated and embedded in Spurr resin. Cells labeled with secondary antibody only served as a control.

The fixed samples were sectioned (90 nm) with a Leica UC6 (Leica Microsystems, Wetzlar, Germany) and viewed on a Hitachi H-7500 (Hitachi, Tokyo, Japan) at 80 kV. Digital images were obtained with a Hamamatsu ORCA-HR digital camera system (Advanced Microscopy Techniques, Woburn, Mass).

### Statistical analysis

Statistical analysis was performed by using 1-way ANOVA or the unpaired *t* test (version 6.04; GraphPad Software, La Jolla, Calif). The α level was set to .05. Unless stated otherwise, only significant changes are indicated in the figures.

## Results

### Human NK cell model of LYST deficiency mimics the cellular phenotype of CHS

**Fig 1:**
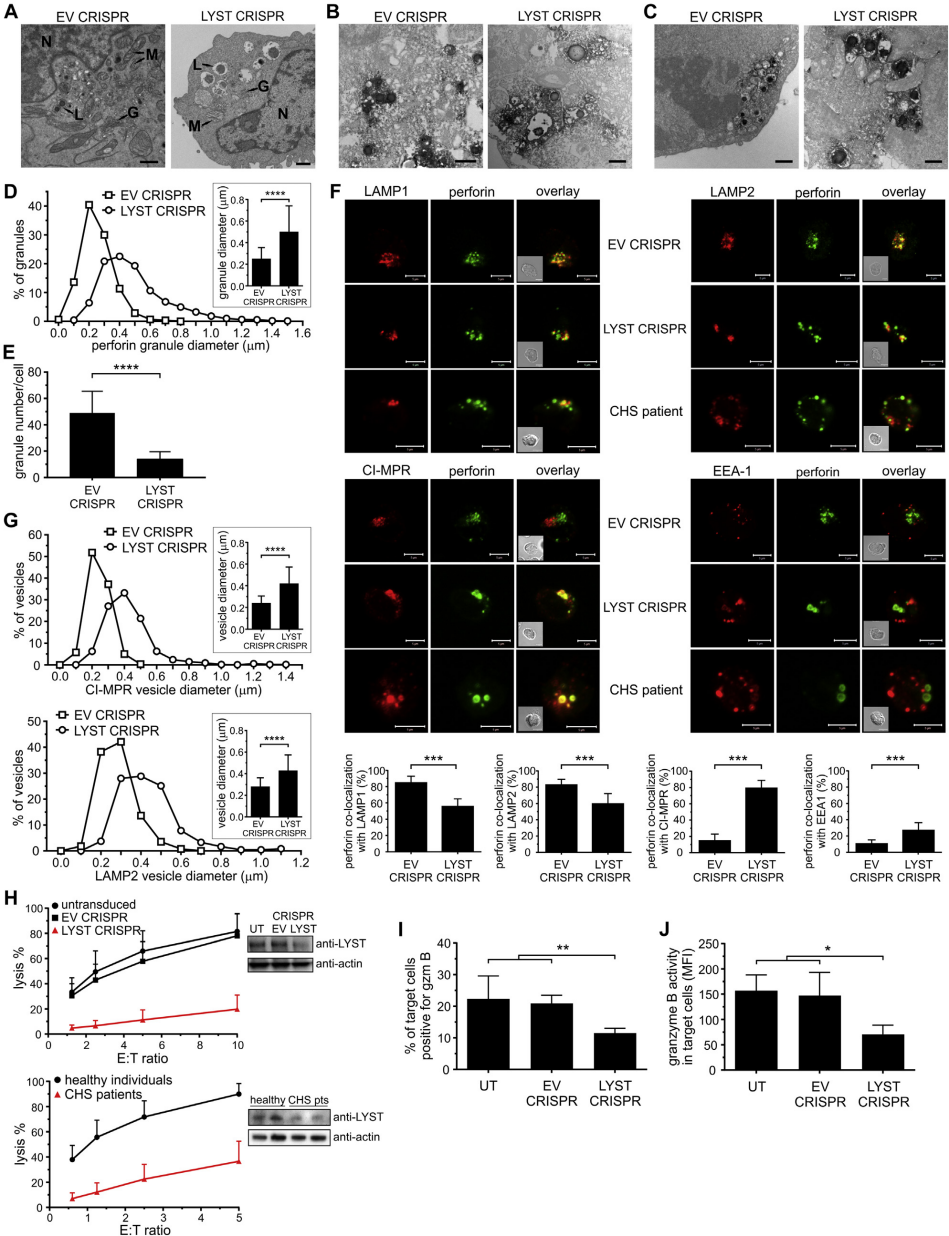
Human NK cell line model of *LYST* deficiency mirrors CHS cell phenotype. **A-G,** Cellular phenotype of *LYST*-deficient NK cells. Fig 1, *A*, Transmission electron microscopy *(TEM)* images. *G*, Golgi apparatus; *L*, lysosomes; *M*, mitochondria; *N*, nucleus. *Scale bars* = 1 μm. Fig 1, *B* and *C*, Immunotransmission electron microscopy *(Immuno-EM)* of LAMP1 or perforin, respectively. *Scale bars* = 800 nm. Fig 1, *D*, Frequency distribution of perforin-positive granule sizes. *Inset* shows the average perforin-positive granule diameter. Fig 1, *E*, Average number of perforin granules. Fig 1, *F*, NK cells stained with antibodies against perforin *(green)* and different vesicular compartments *(red)*: LAMP1 (late endosome/lysosomes), LAMP2 (lysosomes), CI-MPR (post–trans-Golgi network transport vesicles), or EEA-1 (early endosomes). *Insets* show differential interference contrast *(DIC)* images. *Scale bars* = 5 μm. Graphs show the percentage of perforin colocalization with vesicular markers. Fig 1, *G*, Frequency distribution of CI-MPR–positive or LAMP2-positive vesicle sizes. *Insets* show the average size of vesicles. **H,** Cytotoxicity of *LYST*-deficient NK cells at different effector/target *(E:T)* ratios. LYST levels were analyzed by using immunoblotting, and actin served as a loading control. **I** and **J,** Delivery of granzyme B from NK cells. The increase in granzyme B substrate fluorescence in target cells was monitored by using flow cytometry (see Fig E2, *A*). Fig 1, *I*, Percentage of target cells positive for granzyme B activity. Fig 1, *J*, Mean intensity of granzyme B substrate fluorescence in target cells. Data are shown as means + SDs. *P < .05, **P < .01, ***P < .001, and ****P < .0001, unpaired *t* test (Fig 1, *D*, *E*, and *G*) or 1-way ANOVA (Fig 1, *I* and *J*). Fig 1, *D* and *E*, n = 50 to 90 cells from 4 experiments; Fig 1, *F*, n = 18 cells from 2 experiments; Fig 1, *G*, n = 25 to 30 cells from 2 experiments; Fig 1, *H-J*, data from 4 experiments.

One of the major impediments in understanding rare human disorders, such as CHS, is the restricted availability of patient samples. To overcome this limitation, we set out to create a human cell model of CHS using the CRISPR system to facilitate genome editing at the region encoding the *LYST* ARM/HEAT domain. Disruption of the *LYST* gene in a human NK cell line, NK92mi, resulted in generation of a cellular phenotype indistinguishable from that of NK cells from patients with CHS with *LYST* ARM/HEAT domain mutations (Fig 1).

**Fig E1:**
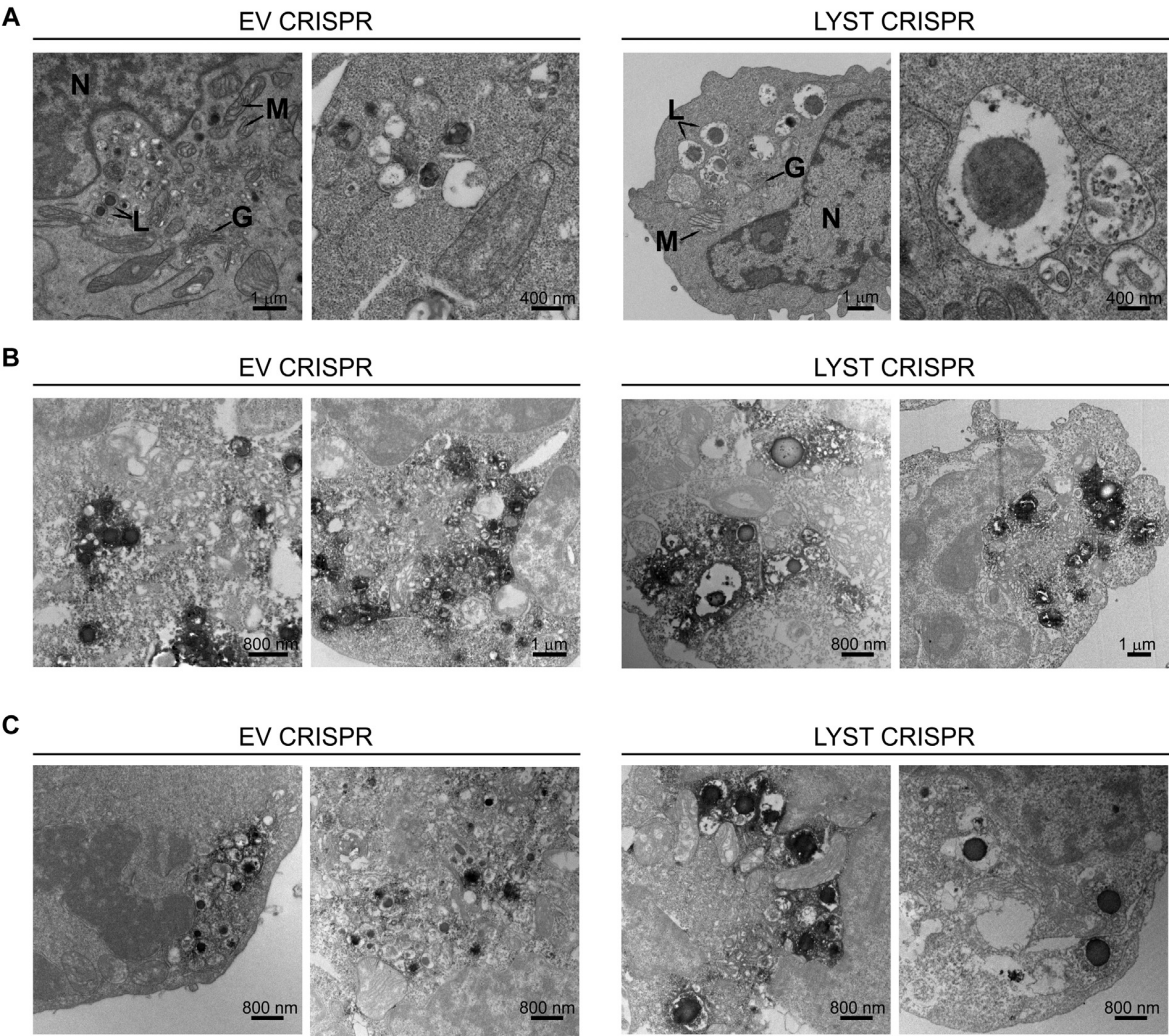
Cellular phenotype of *LYST*-deficient NK cells. **A,** Transmission electron microscopy *(TEM)* images illustrating the cellular ultrastructure of NK92mi cells transduced with EV or LYST CRISPR constructs. *G*, Golgi apparatus; *L*, lysosomes; *M*, mitochondria *N*, nucleus. **B** and **C,** Immunotransmission electron microscopy *(Immuno-EM)* of LAMP1 (Fig E1, *B*) or perforin (Fig E1, *C*) in EV CRISPR– or LYST CRISPR–transduced NK92mi cells. Images in the first columns in Fig E1, *A-C*, are identical to the images shown in Fig 1, *A-C*, and are shown enlarged for visualization purposes; images in the second column provide additional examples. Scale bar scales are indicated in the images.

In contrast to NK92mi cells transduced with empty vector (EV CRISPR), LYST CRISPR NK92mi cells were characterized by visibly enlarged lysosomes, as evidenced by transmission electron microscopy and immunoelectron microscopy (Fig 1, *A-C*, and see Fig E1 in this article's Online Repository at www.jacionline.org). In LYST CRISPR cells more than 90% of perforin-positive lytic granules were equal to or larger than 300 nm, with an average diameter of 502 nm, whereas in normal NK92mi cells 85% of granules were 300 nm or smaller, averaging 252 nm (Fig 1, *D*). In addition, LYST CRISPR NK cells displayed severely reduced numbers of lytic granules (Fig 1, *E*). The distribution of lytic granule sizes and their amount closely resembled those observed for NK cells from patients with CHS.^33^ As in NK cells from patients with CHS, perforin-positive granules in LYST CRISPR cells displayed defective acquisition of several endolysosomal markers (Fig 1, *F*). In EV CRISPR NK92mi cells perforin-containing granules were positive for the lysosomal markers LAMP1 and LAMP2, exhibited a minimal colocalization with CI-MPR–positive transport vesicles, and were distinct from EEA-1–positive early endosomes, as typically observed in normal NK cells.^33^ However, LYST CRISPR NK92mi cells contained granules that were positive for perforin but negative for LAMP1 or LAMP2 and *vice versa*, with nearly all the granules positive for CI-MPR and many acquiring low levels of EEA-1, like NK cells from patients with CHS (Fig 1, *F*). As expected, LAMP2- and CI-MPR–positive vesicles in LYST CRISPR cells were markedly increased in diameter and showed a size distribution similar to perforin-positive granules (Fig 1, *G*).

**Fig E2:**
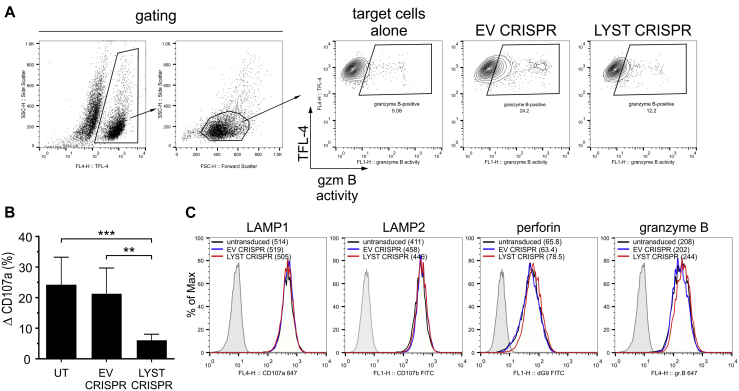
Degranulation ability of *LYST*-deficient NK cells. **A,** Delivery of granzyme B to target cells. 721.221 target cells were labeled with TFL-4 and a fluorogenic substrate of granzyme B and then mixed with EV or LYST CRISPR NK92mi cells. The increase in substrate fluorescence in TFL-4–positive target cells, indicating the activity of granzyme B, was monitored by using flow cytometry. *Dot plots* show gating strategies used and illustrate an example of the result. **B,** Assessment of degranulation. NK92mi cells, either untransduced *(UT)* or transduced with either EV or LYST CRISPR, were incubated alone or with 721.221 target cells for 60 minutes at 37°C. Cells were stained with anti-CD56 and anti-CD107a antibodies, and cell-surface expression of CD107a was determined by using flow cytometry. Change in CD107a cell-surface expression (ΔCD107a) was calculated as the percentage of CD107a-positive activated NK92mi cells after subtraction of the percentage of CD107a-positive NK92mi cells from the nonstimulated population. The graph shows mean values + SDs, as determined from 5 separate experiments. **P < .01 and ***P < .001, 1-way ANOVA. **C,** Intracellular levels of lysosomal proteins. NK92mi cells, either untransduced or transduced with EV or LYST CRISPR constructs, were fixed, permeabilized, and then stained with either anti–LAMP1–Alexa Fluor 647, anti-LAMP2–fluorescein isothiocyanate *(FITC)*, anti-perforin–FITC, or anti–granzyme B–Alexa Fluor 647 antibodies. Cells were then analyzed by using flow cytometry for total levels of the indicated proteins. Histograms show a typical result from 2 independent experiments. *Numbers in parentheses* indicate median fluorescence intensity values for indicated protein staining.

Both LYST CRISPR NK92mi cells and NK cells from patients with CHS displayed severely inhibited cytotoxicity (Fig 1, *H*) that correlated with defective degranulation and granzyme B delivery to target cells (Fig 1, *I*, and see Fig E2, *A* and B, in this article's Online Repository at www.jacionline.org), despite normal levels of perforin, granzyme B, and LAMP proteins (see Fig E2, *C*), which is in line with previous data.^33^, ^46^ Importantly, although LYST CRISPR NK92mi cells were able to deliver some granzyme B to target cells (Fig 1, *I*, and see Fig E2, *A*), the activity of granzyme B in target cells was significantly decreased (Fig 1, *J*). Thus the newly generated human NK cell model faithfully reproduced the reported aspects of the CHS cellular phenotype characteristic for *LYST* mutations within the region encoding the ARM/HEAT domain^33^ and confirmed that LYST is involved in regulation of cytotoxic granule size and exocytosis, as well as maintenance of endolysosomal compartment integrity.

### Enlarged lytic granules in *LYST*-deficient NK cells are functional

**Fig 2:**
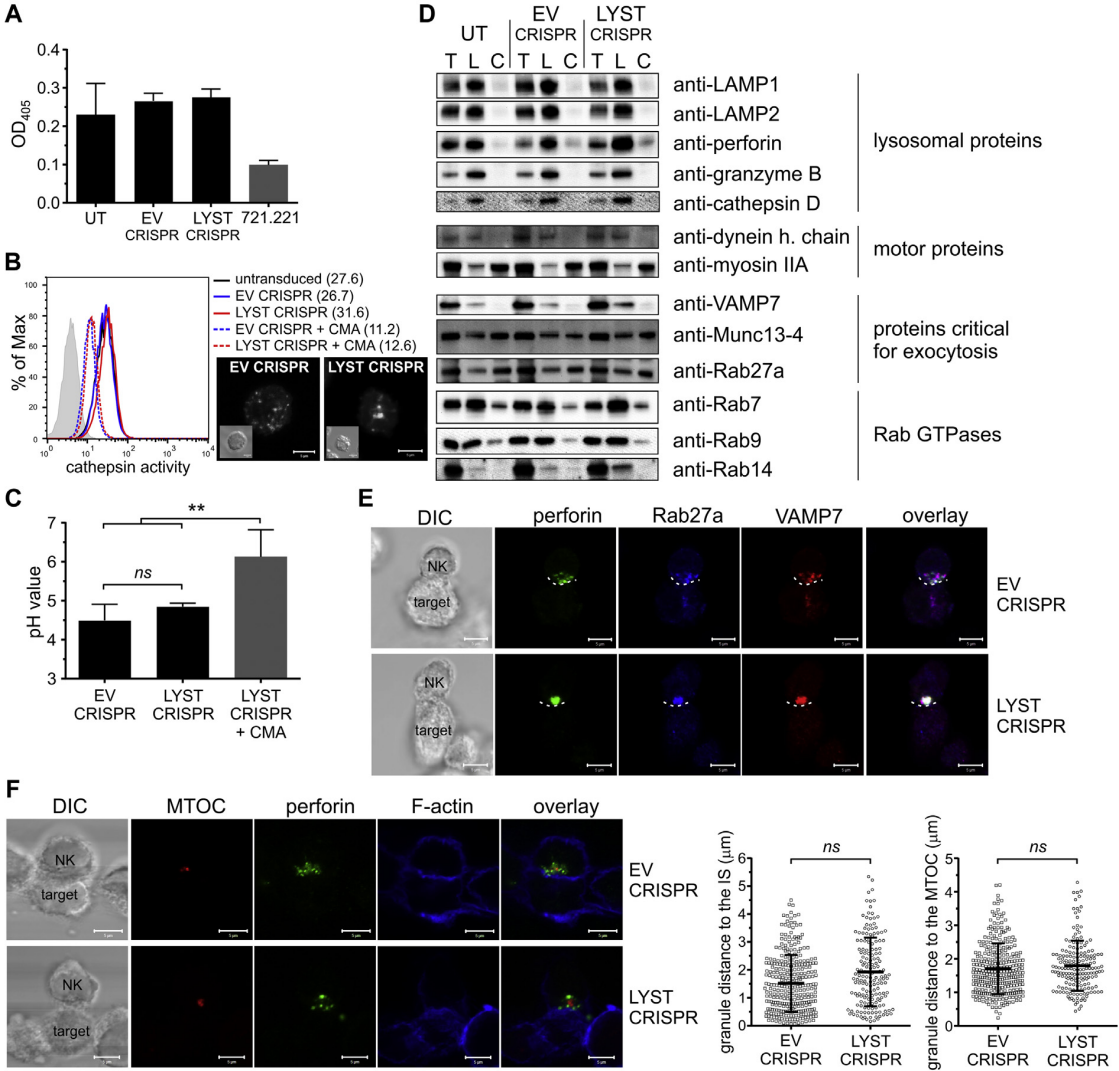
Enlarged lytic granules in *LYST*-deficient NK cells are functional. **A,** Granzyme B activity in total cell lysates. Granzyme B–negative 721.221 cells served as a control. **B,** Proteolytic activity of cathepsin B evaluated by using flow cytometry. Concanamycin A–treated cells served as a negative control. Numbers indicate median fluorescence intensity values. Confocal images show visualization of cathepsin activity in the cells; *insets* indicate differential interference contrast *(DIC)* images. *Scale bars* = 5 μm. **C,** Lysosomal pH. pH values were determined, as described in the Methods section. LYST CRISPR cells treated with concanamycin A were used as a control. **D,** Lytic granule analysis. The presence of proteins in the total cell lysate *(T)*, purified lysosomal fraction *(L)*, and cytoplasmic fraction *(C)* was assessed by means of immunoblotting with antibodies specific for the indicated proteins. Each *lane* was loaded with 1 μg of protein material. **E** and **F,** Perforin polarization to the immunologic synapse. Cells were stained with antibodies against perforin *(green)*, Rab27a *(blue)*, and VAMP7 (*red*; Fig 2, *E*) or with phalloidin (F-actin marker; blue) and antibodies against pericentrin (MTOC marker; red) and perforin (green; Fig 2, *F*). The *dashed line* in Fig 2, *E*, shows the position of the immunologic synapse. *DIC*, Differential interference contrast. *Scale bars* = 5 μm. Graphs in Fig 2, *F*, show the mean distance between perforin-positive granules and the immunologic synapse or MTOC. Graphs show mean values with SDs. **P < .01, 1-way ANOVA (Fig 2, *C*) or the unpaired *t* test (Fig 2, *F*). *ns*, Not significant. Fig 2, *A*, *C*, and *D*, data from 3, 4, or 2 experiments, respectively; Fig 2, *F*, n = 18 conjugates for each cell group.

Having established that LYST CRISPR NK92mi cells are a valid model of the CHS cellular phenotype, we next tested whether the impaired cytotoxic potential of such NK cells could be related to problems with lytic granule function. Analysis of granzyme B and cathepsin proteolytic activity revealed no difference in lysosomal enzyme activity between LYST CRISPR and control cells (Fig 2, *A* and *B*). Lysosomal pH in EV CRISPR and LYST CRISPR NK92mi cells was estimated as 4.5 and 4.8, respectively (Fig 2, *C*), which is in the pH range typical for lysosomes.^47^ Disruption of lysosomal acidification with concanamycin A^48^ resulted in inhibition of cathepsin enzymatic activity and a substantial increase in lysosomal pH (Fig 2, *B* and *C*).

Next, we isolated lytic granules from control and LYST CRISPR cells to examine whether the large granules had altered levels of proteins essential for lytic granule exocytosis. Levels of the major lysosome-associated proteins (LAMP1 and LAMP2) and lytic proteins (perforin and granzyme B), as assessed by using Western blotting, were not changed in LYST CRISPR cells (Fig 2, *D*), which is in line with flow cytometric data (see Fig E2, *C*). Dynein and myosin IIA, 2 motor proteins critical for lytic granule polarization and secretion,^49^, ^50^ were recruited normally to the granules in LYST CRISPR NK cells (Fig 2, *D*). Similarly, Rab27a, Munc13-4, and VAMP7, which are essential for lytic granule docking, priming, and fusion with the plasma membrane,^51^, ^52^, ^53^, ^54^ were present on lytic granules in *LYST*-deficient cells at levels comparable with those of untransduced or EV CRISPR–transduced NK92mi cells (Fig 2, *D* and *E*). Moreover, the large granules in LYST CRISPR cells clustered around the MTOC and polarized to the immunologic synapse similar to normal lytic granules (Fig 2, *F*), indicating that NK cell activation and early stages in the assembly of the immunologic synapse are not affected by LYST deficiency.

**Fig 3:**
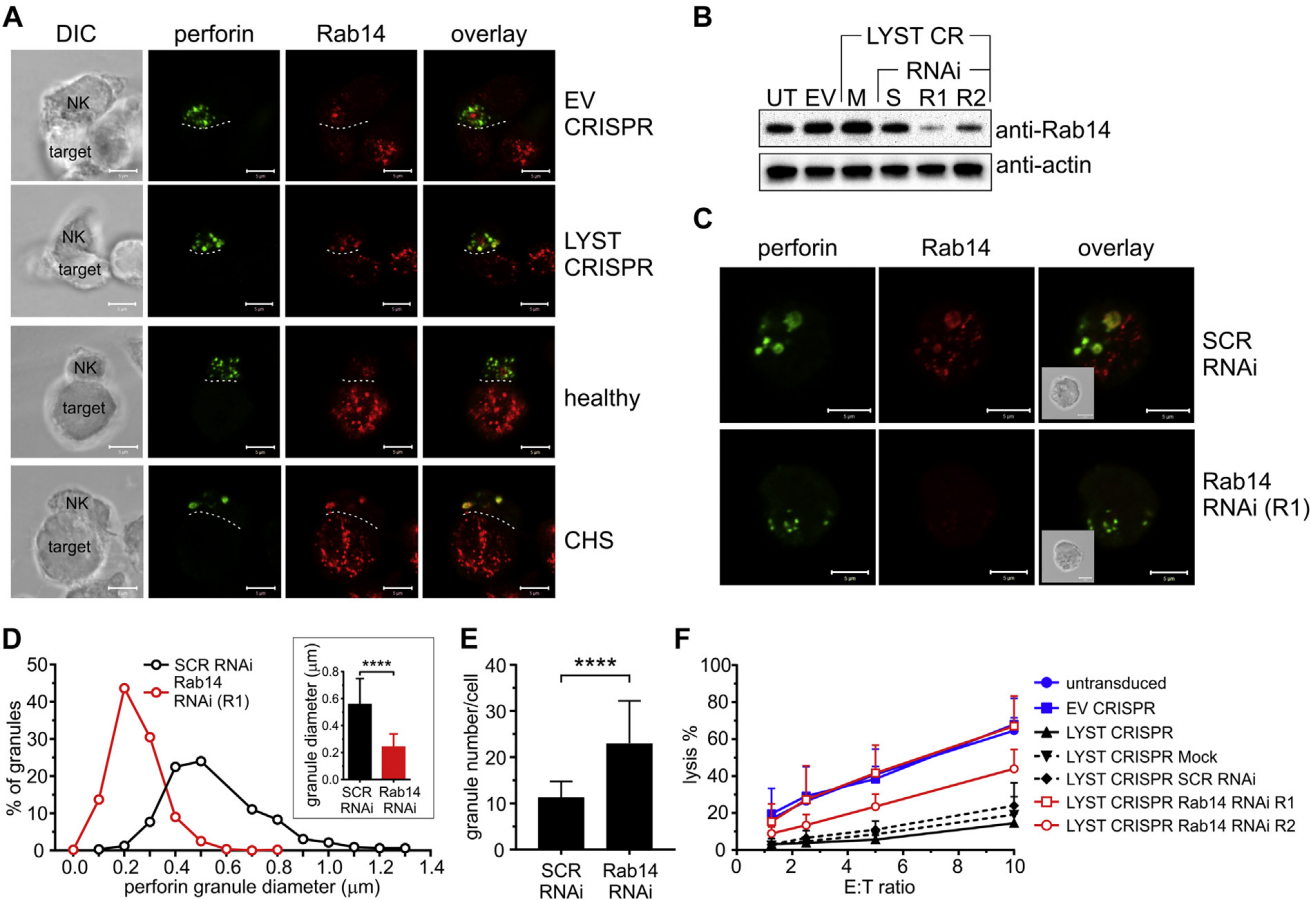
Rab14 silencing in *LYST*-deficient NK cells restores the normal size of lytic granules and cytotoxicity. **A,** Rab14 localization. The indicated NK cells were stained with antibodies against perforin *(green)* and Rab14 *(red)*. The *dashed line* shows the position of the immunologic synapse. *Scale bars* = 5 μm. *DIC*, Differential interference contrast. **B,** Rab14 silencing. LYST CRISPR NK92mi cells were mock transduced *(M)* or transduced with scramble RNAi *(S)* or 2 Rab14 RNAis (*R1* and *R2*). Protein levels of Rab14 were analyzed by means of immunoblotting; actin served as a loading control. *EV*, Empty vector CRISPR-transduced cells; *UT*, untransduced cells. **C,** LYST CRISPR cells transduced with scrambled *(SCR)* RNAi or Rab14 RNAi *(R1)* were stained for perforin *(green)* and Rab14 *(red)*. *Insets* show DIC images. *Scale bars* = 5 μm. **D,** Frequency distribution of perforin-positive granule sizes; *inset* shows the average diameter of perforin-positive granules. **E,** Average number of perforin granules after transduction with the indicated RNAi. *Error bars* in Fig 3, *D* and *E*, indicate SDs. ****P < .0001, unpaired *t* test. N = 28 (SCR RNAi) or 26 (Rab14 RNAi) cells from 2 experiments. **F,** Cytotoxicity of the indicated cells at different effector/target (E:T) ratios. Untransduced and EV CRISPR–transduced NK92mi cells served as controls. Graph shows mean values + SDs determined from 3 experiments.

**Fig E3:**
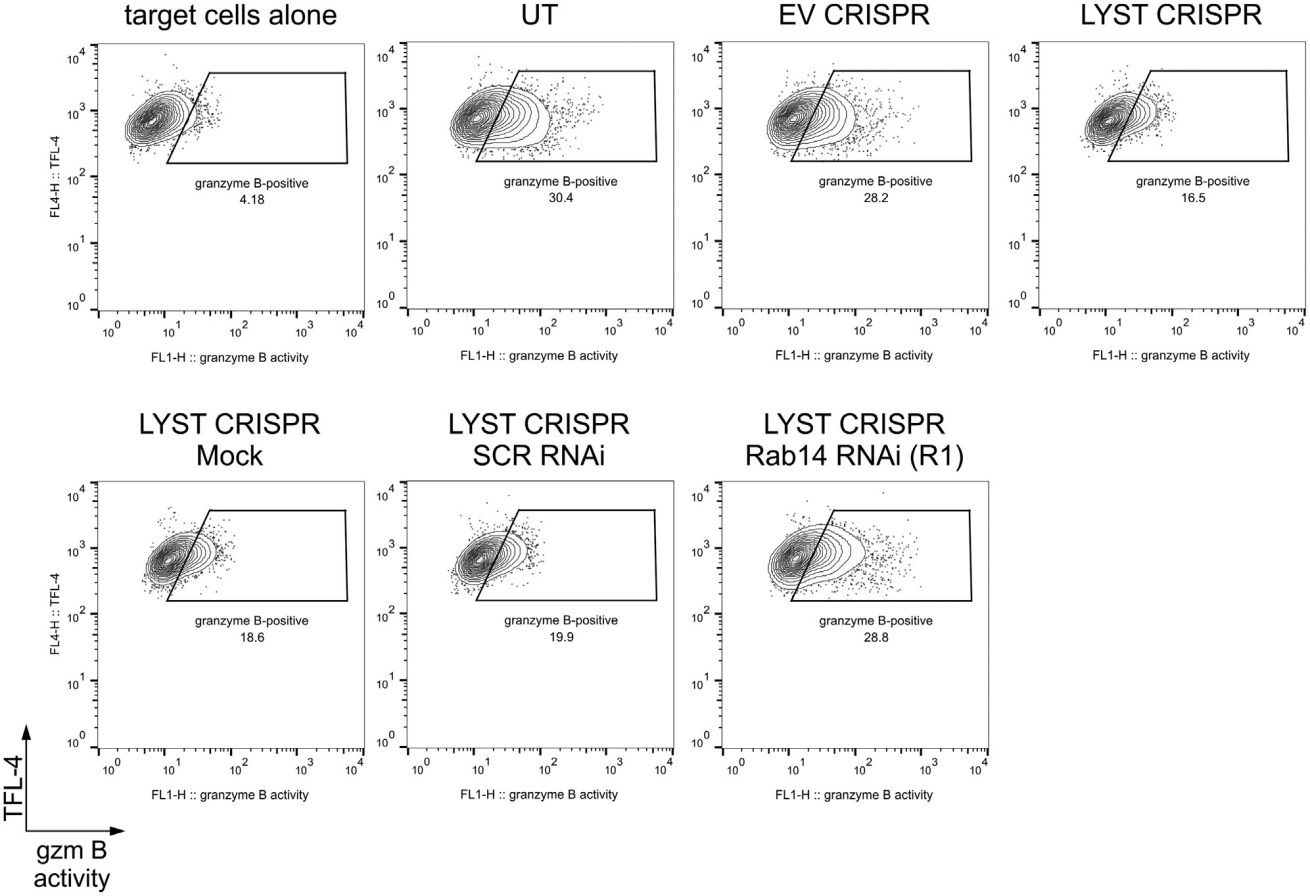
Changes in degranulation ability of *LYST*-deficient NK cells after Rab14 silencing. 721.221 target cells were labeled with TFL-4 and a fluorogenic substrate of granzyme B and then mixed with untransduced *(UT)*, EV, or LYST CRISPR NK92mi cells. The increase in substrate fluorescence in TFL-4–positive target cells, indicating delivery of granzyme B *(gzm B)* to target cells, was monitored by using flow cytometry. *Dot plots* show gating strategy used and illustrate an example of the result.

We noticed that lytic granules from *LYST*-deficient NK cells had increased levels of Rab14 (Fig 2, *D*, and Fig 3, *A*). Because Rab14 has been postulated to act as a LYST antagonist and regulate the size of lysosome-related organelles,^55^ we silenced Rab14 expression in LYST CRISPR cells (Fig 3, *B*). Disruption of Rab14 expression restored the normal size of lytic granules in *LYST*-deficient cells (Fig 3, *C* and *D*). Silencing of Rab14 also resulted in a 2-fold increase in lytic granule numbers in LYST CRISPR cells (Fig 3, *E*), restoring the granule amount to half the normal level (Fig 1, *E*). Importantly, silencing of Rab14 restored the ability of *LYST*-deficient cells to deliver granzyme B to target cells (see Fig E3) and, consequently, their cytolytic capability (Fig 3, *F*). These results suggest that the increased size of lytic granules could be a major factor underlying defective cytotoxicity of NK cells from patients with CHS.

### Cortical actin limits the exocytosis of enlarged granules in *LYST*-deficient NK cells

To be secreted, lytic granules in NK cells need to navigate through the layer of cortical actin at the immunologic synapse.^10^, ^11^, ^44^, ^45^, ^56^, ^57^ Therefore we investigated whether the actin meshwork at the synapse could interfere with the exocytosis of large lytic granules in *LYST*-deficient NK cells.

**Fig E4:**
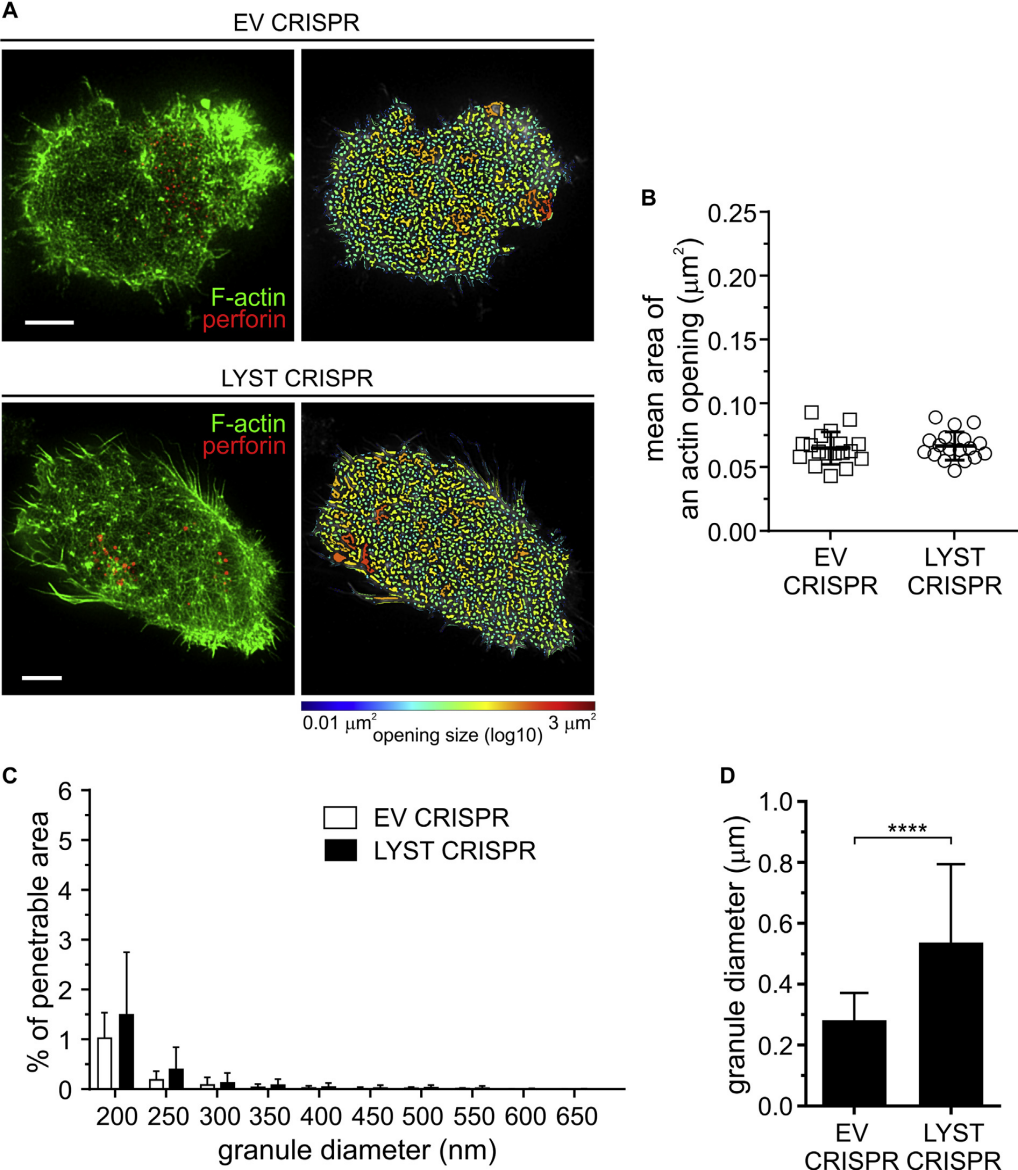
Membrane-proximal actin meshwork at synapses formed through engagement of lymphocyte function–associated antigen 1 *(LFA-1)*. **A,** EV or LYST CRISPR NK92mi cells were incubated on cover slips coated with ICAM-1, stained with anti-perforin antibody (red) and phalloidin (green), and visualized by using STED microscopy. *First column*, Super-resolution images of F-actin meshwork and lytic granules proximal to the plasma membrane. *Scale bars* = 5 μm. *Second column*, Openings among F-actin at the synapse shown as heat maps; the smallest holes (0.01 μm^2^) are indicated in blue, and the largest (approximately 3 μm^2^) are indicated in red. **B,** Quantification of the average size of actin meshwork openings at the synapse center for the indicated NK92mi cells stimulated as in Fig E4, *A*. The graph represents means ± SDs (n = 18 cells per condition from 3 independent experiments). **C,** Quantification of the proportion of the synapse area predicted to be penetrable by vesicles of 200 to 800 nm in diameter for the same cells as in Fig E4, *B*. The graph shown means + SDs (n = 18 cells per condition from 3 independent experiments). There was no significant change in the mean area of actin openings or the percentage of actin meshwork permeability between EV and LYST CRIRPS cells. **D,** Size of lytic granules. EV or LYST CRISPR NK92mi cells were labeled and visualized as in Fig E4, *A*, and the perforin-positive vesicles were recorded in 3 dimensions (x-y-z plane). *Bar graph* shows the average granule diameter in the indicated cells; *error bars* indicate SDs. ****P < .0001, unpaired *t* test.

Visualization of F-actin at the synapse revealed similar organization of the cortical actin between EV CRISPR and LYST CRISPR NK92mi cells. Ligation of lymphocyte function–associated antigen 1 integrin by ICAM-1 triggered lytic granule polarization, along with formation of a dense F-actin mesh at the synapse in both cell types, with small openings that were not penetrable to polarized granules (see Fig E4 in this article's Online Repository at www.jacionline.org). This confirmed no fundamental defects in granule translocation and cortical actin remodeling at the immunologic synapse because of loss of LYST function.

**Fig 4:**
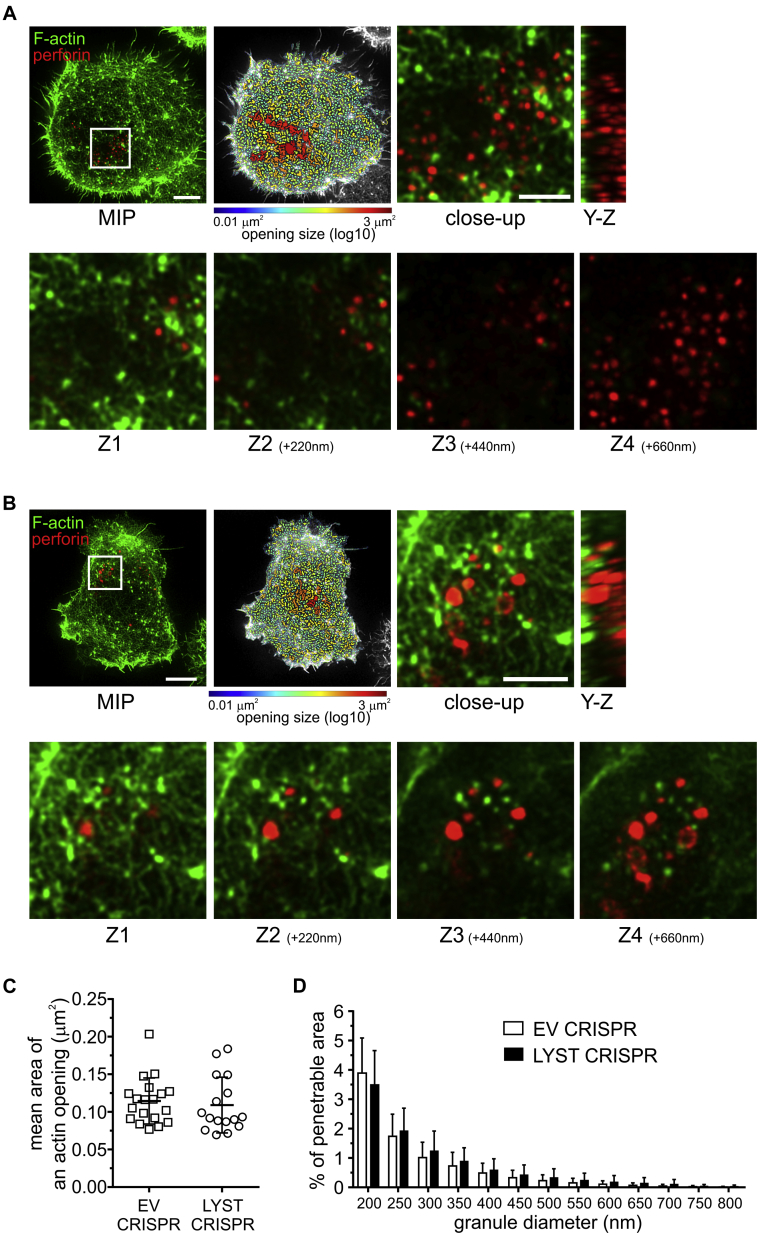
Openings in the cortical actin meshwork at the immunologic synapse are not permissive for enlarged granules in *LYST*-deficient NK cells. **A** and **B,** EV (Fig 4, *A*) or LYST CRISPR NK92mi (Fig 4, *B*) cells forming contacts on cover slips coated with ligands for lymphocyte function–associated antigen 1 *(LFA-1)* and NKG2D, stained with anti-perforin antibody *(red)* and phalloidin *(green)*, and visualized with STED microscopy. The images show F-actin meshwork and lytic granules proximal to the plasma membrane (contact site). *Scale bars* = 5 μm. Openings among actin filaments at the synapse are shown as heat maps. Close-up images show the area of the synapse containing lytic granules (*white rectangles* in the leftmost images). *Scale bars* = 2 μm. Y-Z plane projections show the position of lytic granules relative to the actin filaments. The relationship between F-actin and lytic granules is illustrated by optical sections (Z1-Z4) acquired sequentially every 220 nm and progressively distal to the contact site. **C,** Quantification of the average size of actin meshwork openings at the synapse center for cells stimulated as in Fig 4, *A* and *B*. **D,** Quantification of the proportion of the actin meshwork at the synapse area predicted to be penetrable by 200- to 800-nm-diameter vesicles, for the same cells as Fig 4, *C*. Graphs represent means, and *error bars* indicate SDs (n = 18 cells per condition from 3 experiments). There was no significant difference in actin opening sizes or actin meshwork permeability between EV and LYST CRIRPS cells. *MIP*, Maximum intensity projection.

Coligation of lymphocyte function–associated antigen 1 and an activation receptor, NKG2D, resulted in substantial remodeling of F-actin at the synapse formed by EV or LYST CRISPR NK cells and generation of hypodense actin areas in the central part of the synapse (Fig 4, *A* and *B*). Whereas most normal-sized granules in EV CRISPR NK92mi cells were found to fit the size of the opening in actin-sparse areas, in LYST CRISPR cells the large granules were frequently seen above the actin filaments, with only a few able to fit into the openings in the actin mesh (Fig 4, *A* and *B*). No significant differences in F-actin rearrangements were observed between EV and LYST CRISPR NK cells, as evidenced by the comparable size of actin openings and the percentage of vesicle-penetrable area in the actin meshwork (Fig 4, *C* and *D*), confirming that LYST does not play a critical role in activation of NK cells or in the early stages of the immunologic synapse assembly. The majority of actin openings were determined to be accessible to granules less than 300 nm in diameter (Fig 4, *C* and *D*). In EV CRISPR NK cells granules could fit through such openings because their mean size was 252 nm (Fig 1, *D*, and 4, *D*). However, the large granules in LYST CRISPR cells were unable to access those hypodense areas because less than 1% of the actin meshwork at the synapse was permissive for granules with an average diameter of 502 nm (Fig 1, *D*, and 4, *D*). These results suggest that cortical actin could act as a mechanical hindrance for exocytosis of large lytic granules.

### Reduction of actin meshwork density restores degranulation of *LYST*-deficient NK cells

To test the idea that the subsynaptic actin meshwork is a barrier to secretion of large granules, we investigated whether small molecules interfering with actin polymerization could potentiate the exocytosis of lytic granules from LYST CRISPR NK cells.

**Fig 5:**
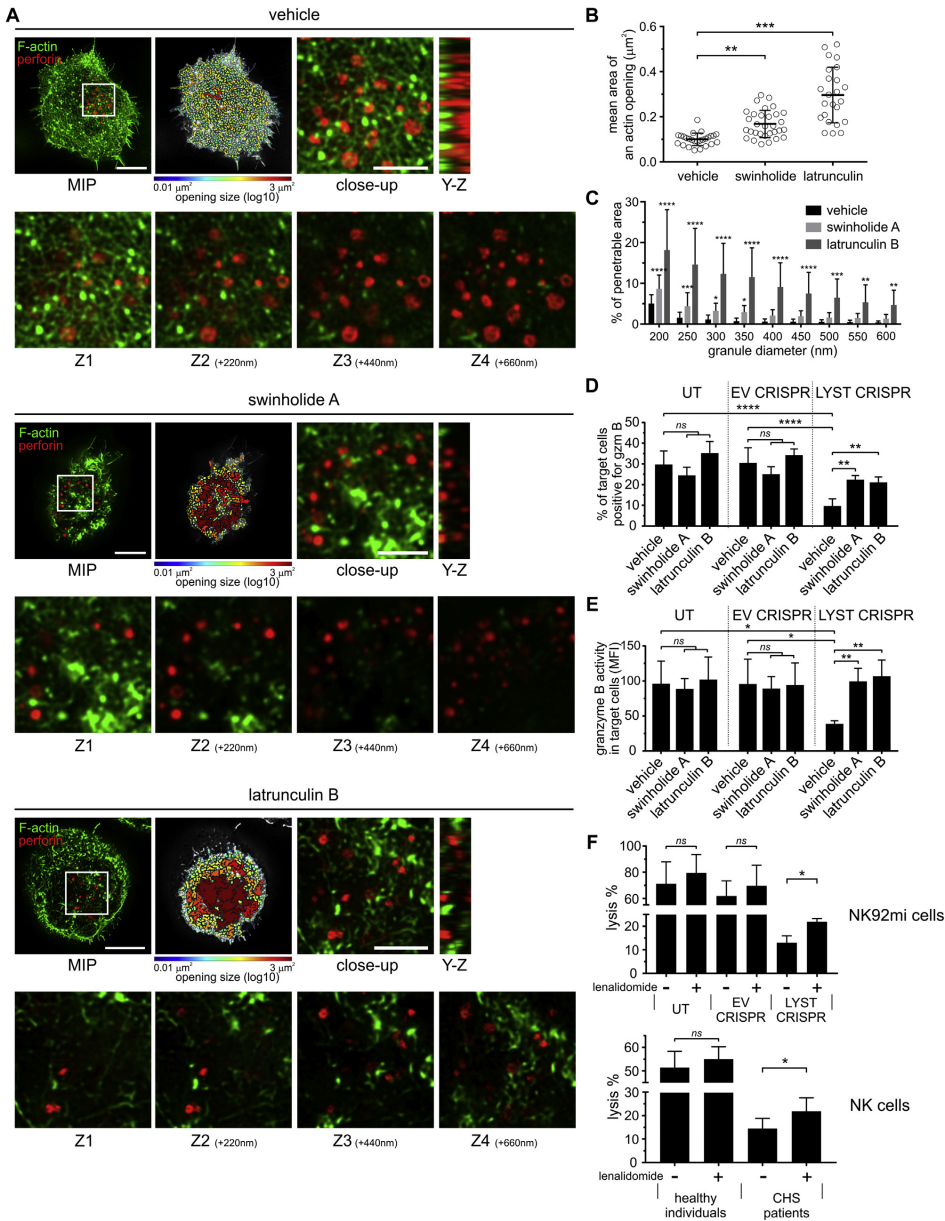
Modulation of cortical actin meshwork density restores degranulation of *LYST*-deficient NK cells. **A,** Images of F-actin *(green)* and lytic granules *(red)* proximal to the contact site formed by LYST CRISPR cells and treated with dimethyl sulfoxide (vehicle), swinholide A, or latrunculin B. *Scale bars* = 5 μm. Openings in the actin meshwork are shown as heat maps. Close-up images show the synapse area containing lytic granules (*white rectangles* in the leftmost images). *Scale bars* = 2 μm. Position of lytic granules relative to the actin filaments is illustrated by Y-Z plane projections and optical sections acquired every 220 nm from the contact site (Z1-Z4). *MIP*, Maximum intensity projection. **B** and **C,** Size of actin openings and penetrability of the actin meshwork at the synapse for cells treated and stimulated as in Fig 5, *A*. **D** and **E,** Granzyme B delivery from NK92mi cells, untransduced *(UT)* or transduced with EV or LYST CRISPR, and pretreated with dimethyl sulfoxide (vehicle), swinholide A, or latrunculin B. The change in granzyme B substrate fluorescence in target cells was monitored by using flow cytometry (see Fig E5). Fig 5, *D*, Percentage of target cells positive for granzyme B. Fig 5, *E*, The intensity of granzyme B substrate fluorescence in target cells. **F,** Cytotoxicity of NK cells pretreated with dimethyl sulfoxide *(—)* or lenalidomide *(+)*. The percentage of target cell lysis is shown at a 10:1 and 2:1 effector/target *(E:T)* ratio for NK92mi and *ex vivo* NK cells, respectively. Graphs show mean values with SDs determined from 4 to 5 (Fig 5, *B-E*) or 2 to 3 (Fig 5, *F*) experiments. *P < .05, **P < .01, ***P < .001, and ****P < .0001, 1-way (Fig 5, *B*, *D*, and *E*) or 2-way (Fig 5, *C*) ANOVA or paired *t* test (Fig 5, *F*).

**Fig E5:**
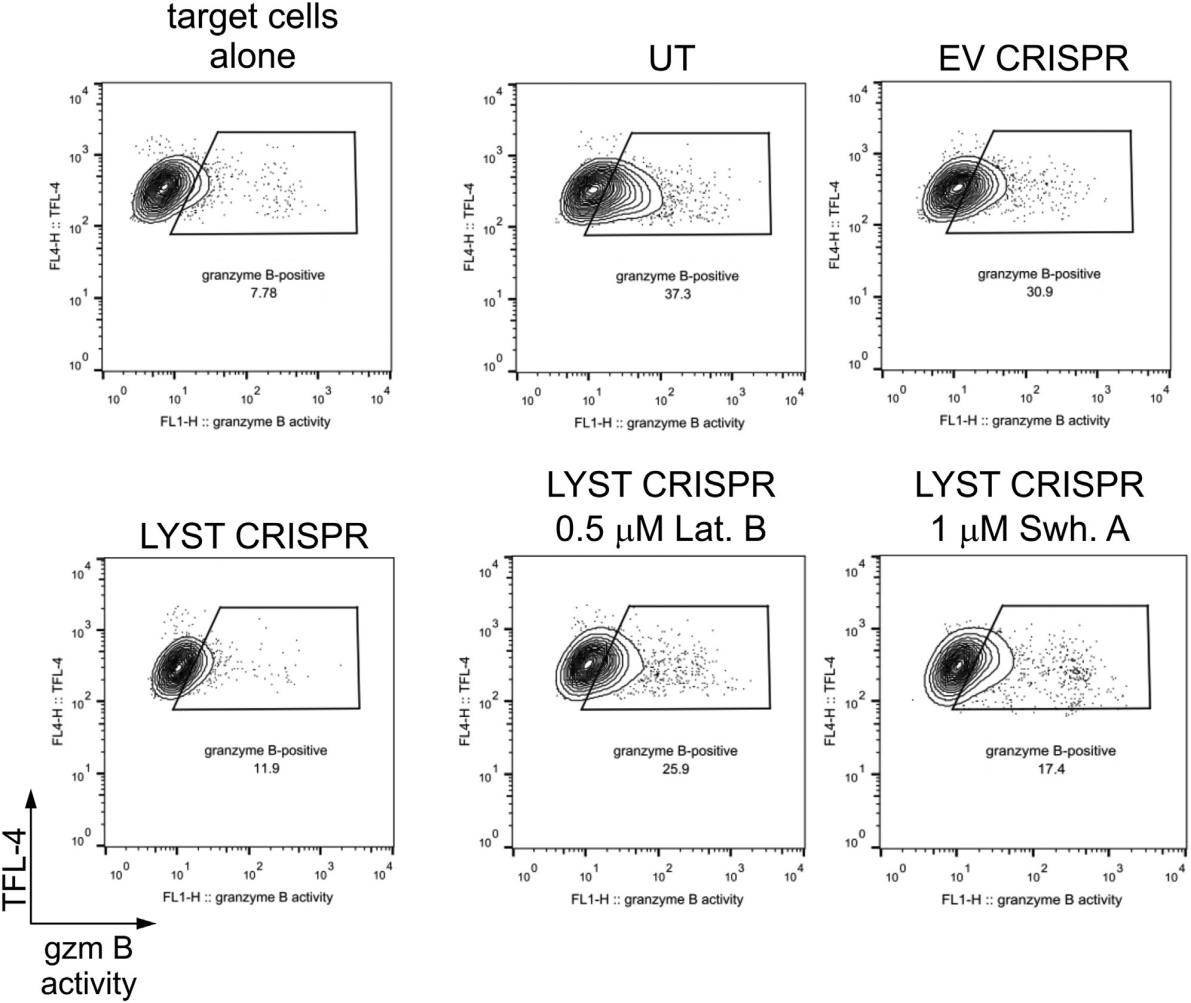
Effects of modulation of actin cytoskeleton density on degranulation of *LYST*-deficient NK cells. NK92mi cells, either untransduced *(UT)* or transduced with EV CRISPR were pretreated with dimethyl sulfoxide; LYST CRISPR NK92mi cells were pretreated with dimethyl sulfoxide, 1 μmol/L swinholide A, or 0.5 μmol/L latrunculin B. Next, cells were mixed with 721.221 target cells and labeled with a fluorogenic substrate of granzyme B *(gzm B)*. The change in substrate fluorescence in target cells was monitored by using flow cytometry. *Dot plots* show gating strategies used and illustrate an example of the result.

**Fig E6:**
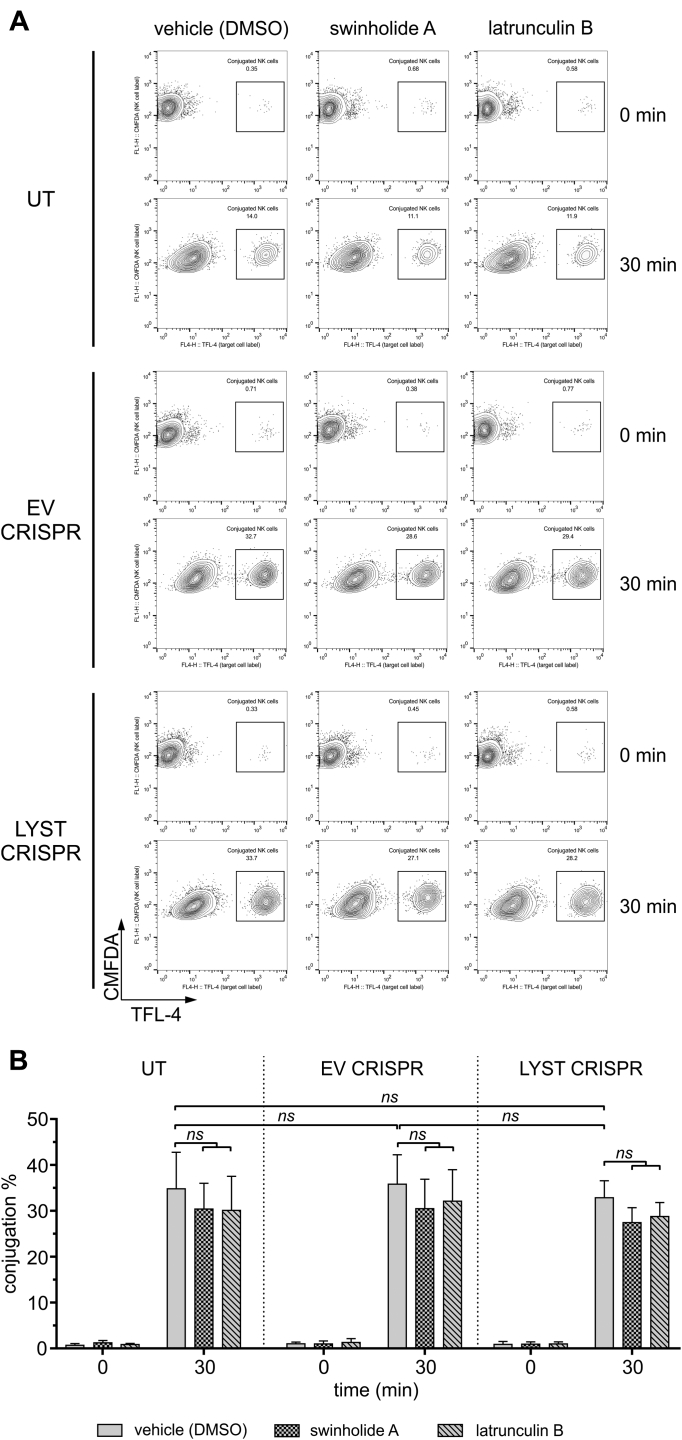
Conjugation of *LYST*-deficient NK cells. NK92mi cells, either untransduced *(UT)* or transduced with EV or LYST CRISPR were stained with CMFDA and then pretreated with dimethyl sulfoxide (*DMSO*; vehicle), 1 μmol/L swinholide A, or 0.5 μmol/L latrunculin B. Next, cells were mixed with TFL-4–labeled 721.221 target cells. Cells were allowed to form conjugates for 30 minutes at 37°C, fixed, and analyzed by using flow cytometry. Conjugates of NK and target cells were determined by measuring the percentage of CMFDA and TFL-4 double-positive cells from the total pool of live CMFDA-positive cells. **A,***Dot plots* show gating strategy used and illustrate an example of the result. **B,** Graph shows conjugation summary data expressed as mean + SD values determined from 4 experiments.

We used latrunculin B, which sequesters monomeric actin, thus inhibiting actin polymerization and promoting depolymerization of F-actin, as well as swinholide A, which severs actin filaments and also prevents actin polymerization through binding of actin dimers.^58^ Compared with vehicle-treated controls, treatment of LYST CRISPR NK cells with swinholide A or latrunculin B induced a significant increase in the penetrability of the actin meshwork and expansion of actin openings at the immunologic synapse, and the large granules were often found in the actin hypodense areas (Fig 5, *A-C*). Moreover, whereas LYST CRISPR NK92mi cells were inefficient in delivering granzyme B to target cells, their treatment with latrunculin B or swinholide A significantly enhanced their ability to deliver granzyme B (Fig 5, *D*, and see Fig E5 in this article's Online Repository at www.jacionline.org). Of note, treatment with latrunculin B or swinholide A slightly reduced NK–target cell conjugation, but there was no difference in the ability of LYST CRISPR NK92mi cells to form conjugates with target cells when compared with normal NK92mi cells (see Fig E6 in this article's Online Repository at www.jacionline.org). The activity of granzyme B in target cells delivered from latrunculin- or swinholide-treated LYST CRISPR cells was comparable with that of normal NK92mi cells (Fig 5, *E*), indicating a substantial increase in the amount of granzyme delivered to target cells when compared with untreated LYST CRISPR cells.

A recent report showed that an immunomodulatory agent, lenalidomide, augments the openings in the cortical actin meshwork at the NK immunologic synapse.^44^ Therefore we tested whether lenalidomide could affect the function of *LYST*-deficient NK cells and provide a new avenue for a potential CHS therapy. Although the treatment effect was small in the *in vitro* conditions tested, lenalidomide partially restored the cytotoxicity of LYST CRISPR NK92mi cells and NK cells from patients with CHS (Fig 5, *F*). Collectively, our data indicate that lytic granules in *LYST*-deficient NK cells are functional and that their physical size impedes their passage through the cortical actin meshwork, thereby impairing NK cell cytotoxic potential.

## Discussion

Progress in understanding and treating CHS has been limited by the lack of tools to investigate the cellular mechanisms affected by loss of LYST function. Using CRISPR/Cas9-mediated genome editing, we generated *LYST*-deficient NK cells that reproduced the cellular phenotype reported for patients with CHS with mutations in the ARM/HEAT domain of *LYST*, including defective cytotoxicity, impaired granzyme delivery to target cells, enlarged lytic granules, and altered integrity of endolysosomal compartments.^33^ Importantly, the CHS cell model allowed us to dissect the relevance of these cellular defects with respect to NK cell function.

LYST has been implicated in regulating endosome, lysosome, and lysosome-related organelle structure, trafficking, and protein sorting.^18^, ^59^, ^60^, ^61^ Loss of LYST function leads to formation of giant lysosomes and lysosome-related organelles.^17^, ^18^, ^62^ These large vesicles often accumulate markers of lysosomes, Golgi-derived transport vesicles, and late, recycling, or even early endosomes.^33^, ^63^ Formation of those enlarged structures does not appear to affect cell physiology,^64^ suggesting that despite inappropriate generation and assembly of secretory lysosomes and their mixed endolysosomal characteristic, the large granules in cells from patients with CHS remain functional. Indeed, we found that the enlarged granules in NK cells from patients with CHS have normal levels and activity of lysosomal enzymes and a pH value characteristic of lysosomes, indicating that their acidification and lysosomal protein processing is not affected. Our results with NK cells, together with data showing that delivery and processing of proteins in the lytic granules in cytotoxic T cells (CTLs), lysosomal degradation of endogenous proteins in fibroblasts, and acidification of enlarged lysosomes in B cells and fibroblasts are unaffected in patients with CHS,^24^, ^65^, ^66^ indicate that the large granules in *LYST*-deficient cells are functional. Thus although LYST is involved in controlling the size and identity of the lysosomal compartments, it does not appear to alter their functional capacity.

Nonetheless, cells from patients with CHS display functional defects, such as impaired exocytosis from melanocytes, platelets, neutrophils, or lymphocytes, which contribute to oculocutaneous albinism, bleeding diathesis, and, importantly, immune dysfunction in patients with CHS.^17^, ^18^, ^24^, ^62^, ^67^ In fact, defective NK cell cytotoxicity is a hallmark of CHS,^26^, ^33^, ^46^, ^68^, ^69^ and impaired lytic function of cytotoxic lymphocytes is thought to underlie the development and progress of life-threatening HLH.^20^, ^21^, ^70^, ^71^ In this regard we found that NK cell activation was not affected by loss of LYST function, as evidenced by accumulation of F-actin at the immunologic synapse and normal polarization of the MTOC to the cell-cell contact site. We observed that NK cells from patients with CHS were able to deliver low amounts of granzyme B to target cells, indicating that defective lytic granule exocytosis underlies the impaired cytotoxic function of NK cells, substantiating previous reports.^33^, ^46^

Lytic granule exocytosis is a complex process requiring several steps and checkpoints.^6^, ^7^ Disruption of function or localization of proteins involved in lytic granule movement, docking, or priming (eg, motor proteins, Rab27a, Munc13-4, and soluble N-ethylmaleimide-sensitive factor activating protein receptor) results in faulty lytic granule exocytosis from cytotoxic lymphocytes.^6^, ^16^, ^70^, ^72^, ^73^ Analysis of the composition of lytic granules isolated from control and LYST CRISPR cells revealed no alterations in the recruitment of essential lytic granule proteins attributable to LYST deficiency. This suggested that the large granules have the protein machinery required for their release,^6^ leading us to pursue an alternative explanation for their defective secretion in patients with CHS.

Lytic granules in NK cells need to traverse through size-restricted gaps in the layer of cortical actin at the immunologic synapse before they can fuse with the plasma membrane and release their contents.^10^, ^11^, ^56^, ^57^ Therefore we hypothesized that the actin meshwork at the synapse could interfere with exocytosis of the large lytic granules in *LYST*-deficient NK cells. Indeed, our data are consistent with the idea that the size of openings in the cortical actin meshwork at the NK cell immunologic synapse is too small for the enlarged granules to pass through. In agreement, reduction of granule size through disruption of Rab14 expression restored cytotoxicity in *LYST*-deficient NK cells. These results not only verify that Rab14 acts as a LYST antagonist in human cells, as in *Dictyostelium* species,^55^ but also confirm that cell activation and lytic granule functionality are normal in NK cells from patients with CHS, and the limited area of actin clearances at the immunologic synapse accessible for the large granules in *LYST*-deficient NK cells restricts their release.

Our results with actin-binding drugs suggest that decreasing the actin cytoskeleton density could help the large granules to reach and fuse with the plasma membrane and successfully release their contents. Thus increasing relaxation of this physical obstruction could provide a means to restore cytolytic function of NK cells from patients with CHS. Because use of actin inhibitors is not feasible in the clinic, we used lenalidomide, an agent previously shown to expand hypodense actin regions at the NK cell immune synapse,^44^ in an attempt to restore NK cell function of patients with CHS. Lenalidomide only partially improved NK cytotoxicity in patients with CHS. This could be because lenalidomide induces openings in the actin filaments permissive for granules less than 350 nm in diameter.^44^ Because the majority of granules in NK cells from patients with CHS are larger than 300 nm (this report and Gil-Krzewska et al^33^), lenalidomide-enhanced hypodense areas at the synapse might remain too restrictive for efficient passage of those large granules. Nevertheless, our results provide proof of concept that restoration of NK cell degranulation and cytotoxicity is possible in patients with CHS. Identification of a compound more efficient than lenalidomide warrants further investigation because it could provide a new avenue for treatment of CHS.

Collectively, our data indicate that in NK cells the problems resulting from LYST deficiency are related to physical hindrances that affect cell functionality. This not only provides insight into understanding the pathology of CHS but also offers a new direction for a therapeutic approach to treat this disease. It remains to be elucidated whether remodeling and decreasing the compactness of the actin cytoskeleton at the synapse would benefit only NK cells or other cells as well. Actin cytoskeleton rearrangements are required for exocytosis from neutrophils, implying that the cortical actin meshwork limits the rate and extent of neutrophil granule exocytosis by controlling granule access to the plasma membrane.^74^ In adrenal chromaffin cells exocytosis occurs preferentially at sites where the actin cortex has gaps,^75^ and increasing the degree of actin polymerization is detrimental to secretory granule exocytosis,^76^ supporting the idea that cortical actin acts as a barrier that hampers fusion. In CTLs cortical actin density is rapidly reduced at the synapse on contact with target cells,^77^ and lytic granules are delivered to actin hypodense areas of the synapse,^78^ suggesting that the actin meshwork could also create a hindrance for the large granule exocytosis in CTLs from patients with CHS.

Of course, factors other than granule size or cortical actin density could be responsible for functional defects observed in CTLs and NK cells in patients with CHS. For instance, the type and/or position of *LYST* mutations are likely to influence the genotype-phenotype correlation and thus the disease manifestations.^22^, ^33^, ^79^, ^80^ Overexpression of exocytic vesicle effectors (ie, Rab27a, Munc13-4, or Slp3) has also been shown to restore degranulation by CTLs from patients with CHS, an effect attributed to the compromised delivery of those effector proteins to lytic granules in CTLs, resulting in a fusion-incompetent state.^63^ This does not exclude the possibility that the physical barrier generated by the actin cytoskeleton contributes to the impaired exocytosis by CTLs seen in patients with CHS. The Rab27a effector Slp1 is necessary to dismantle polymerized cortical actin to facilitate granule exocytosis in neutrophils,^81^ and therefore overexpression of Rab27a and its effectors could improve granule exocytosis by enhancing cortical actin openings. Therefore because actin remodeling decreases actin meshwork density at the CTL immunologic synapse, overexpression of exocytic vesicle effectors is likely to increase the rate, extent, or both of granule fusion with the plasma membrane, thereby promoting degranulation of CTLs.

Restoration of cytotoxic lymphocyte function in patients with CHS would have a tremendous effect on the patients' welfare because it could avert the development of HLH or extend the period of time patients with CHS could wait for a hematopoietic stem cell transplantation required for HLH prevention.

**Key messages**

• Large lytic granules in NK cells from patients with CHS are functional; LYST is involved in controlling the size of the lysosomal compartments but does not appear to alter their capacity to cause cytotoxicity.
• The periodicity of cortical actin limits the exocytosis of enlarged granules in LYST-deficient NK cells, thereby limiting NK cell cytotoxic potential.
• Problems resulting from LYST deficiency are related to physical hindrances that affect cell functionality.
• Restoration of cytotoxic lymphocyte cytolytic function in patients with CHS is possible.
